# Excellent, Lightweight and Flexible Electromagnetic Interference Shielding Nanocomposites Based on Polypropylene with MnFe_2_O_4_ Spinel Ferrite Nanoparticles and Reduced Graphene Oxide

**DOI:** 10.3390/nano10122481

**Published:** 2020-12-10

**Authors:** Raghvendra Singh Yadav, Anju, Thaiskang Jamatia, Ivo Kuřitka, Jarmila Vilčáková, David Škoda, Pavel Urbánek, Michal Machovský, Milan Masař, Michal Urbánek, Lukas Kalina, Jaromir Havlica

**Affiliations:** 1 Centre of Polymer Systems, University Institute, Tomas Bata University in Zlín, Trida Tomase Bati 5678, 760 01 Zlín, Czech Republic; deswal@utb.cz (A.); jamatia@utb.cz (T.J.); kuritka@utb.cz (I.K.); vilcakova@utb.cz (J.V.); dskoda@utb.cz (D.Š.); urbanek@utb.cz (P.U.); machovsky@utb.cz (M.M.); masar@utb.cz (M.M.); murbanek@utb.cz (M.U.); 2 Materials Research Centre, Brno University of Technology, Purkyňova 464/118, 61200 Brno, Czech Republic; kalina@fch.vut.cz (L.K.); havlica@fch.vut.cz (J.H.)

**Keywords:** spinel ferrite, nanocomposites, electromagnetic interference shielding, magnetic loss, dielectric loss

## Abstract

In this work, various tunable sized spinel ferrite MnFe_2_O_4_ nanoparticles (namely MF20, MF40, MF60 and MF80) with reduced graphene oxide (RGO) were embedded in a polypropylene (PP) matrix. The particle size and structural feature of magnetic filler MnFe_2_O_4_ nanoparticles were controlled by sonochemical synthesis time 20 min, 40 min, 60 min and 80 min. As a result, the electromagnetic interference shielding characteristics of developed nanocomposites MF20-RGO-PP, MF40-RGO-PP, MF60-RGO-PP and MF80-RGO-PP were also controlled by tuning of magnetic/dielectric loss. The maximum value of total shielding effectiveness (SE_T_) was 71.3 dB for the MF80-RGO-PP nanocomposite sample with a thickness of 0.5 mm in the frequency range (8.2–12.4 GHz). This lightweight, flexible and thin nanocomposite sheet based on the appropriate size of MnFe_2_O_4_ nanoparticles with reduced graphene oxide demonstrates a high-performance advanced nanocomposite for cutting-edge electromagnetic interference shielding application.

## 1. Introduction

Extensive practice of electronic and communication devices, liberating electromagnetic (EM) waves, generates EM radiation pollution [1]. Electromagnetic interference (EMI) does not only affect the working and life of electronic devices but also is harmful to human health [2]. This noble type of EM radiation pollution delivers a solid motivation to develop efficient EMI shielding materials [3]. Lightweight, thinness and cost efficiency are other additional necessities of high-performance EMI shielding materials for operational applications [4]. Polymer-based EMI shielding composite materials are lightweight, resistant to corrosion, flexible and simple in preparation [5]. The performance of polymer-based EMI shielding materials depends on the intrinsic electrical conductivity, aspect ratio, and concentration of the fillers [6]. Graphene has received considerable attention as nano-fillers due to their excellent electrical and thermal conductivities, and ultrahigh mechanical characteristics [7]. Additionally, spinel ferrite nanoparticles as nanofillers have been established as potential magnetic absorbers due to their outstanding magnetic loss, good stability and cost-effectiveness [8,9].

The particle shape and size of nanoparticles have a vital impact on the microwave absorption and electromagnetic interference shielding characteristics of nanoparticles and their nanocomposites [10]. In recent years, researchers have noticed the influence of particle size on microwave absorption and electromagnetic shielding performance [11]. Yi-Jun Liang et al. [12] noticed the size-dependent microwave absorption performance of Fe_3_O_4_ nanoparticles prepared by the rapid microwave-assisted thermal decomposition method. Niandu Wu et al. [13] observed particle size-dependent microwave absorption characteristics of carbon-coated nickel nanocapsules. A correlation of particle size with electromagnetic parameters can benefit us in better control of electromagnetic interference shielding performance. Our research group [14] also noticed that the particle size of NiFe_2_O_4_ nanoparticles correlates with the electromagnetic interference shielding performance of nanocomposites.

Efficient electromagnetic interference shielding nanocomposite material having a feature of lightweight, flexible and excellent shielding characteristics are highly essential. Here, lightweight, flexible and excellent EMI-shielding nanocomposites with control of magnetic loss/dielectric loss through the control of particle size of MnFe_2_O_4_ spinel ferrite embedded in polypropylene matric with reduced graphene oxide have been developed. Various sized MnFe_2_O_4_ spinel ferrite nanoparticles were synthesized by sonochemical synthesis at different sonication times.

## 2. Materials and Methods

### 2.1. Materials

The reagents manganese nitrate, iron nitrate and sodium hydroxide were procured from Alfa Aesar GmbH and Co KG (Karlsruhe, Germany). Potassium permanganate and graphite flakes were acquired from Sigma-Aldrich, (Munich, Germany). Sodium nitrate was obtained from Lach-Ner (Brno, Czech Republic). The utilized polypropylene (Vistamaxx 6202) was procured from Exxon Mobil (Machelen, Belgium). The reducing agent Vitamin C (Livsane) was obtained from Dr. Kleine Pharma GmbH, (Bielefeld, Germany).

### 2.2. Preparation of Nanoparticles

**Figure 1:**
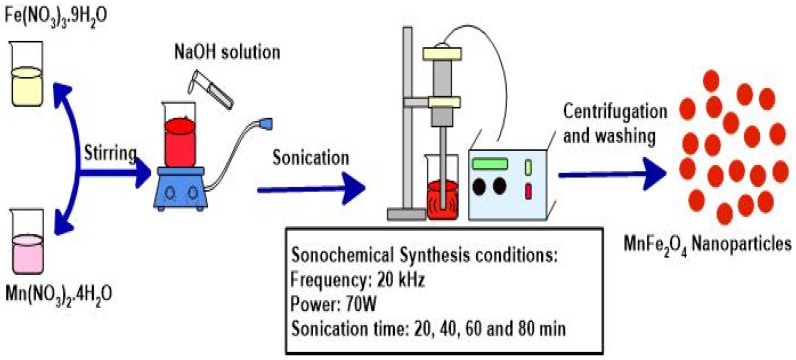
Schematic illustration of the preparation of MnFe_2_O_4_ nanoparticles by the sonochemical synthesis method.

Various sized MnFe_2_O_4_ spinel ferrite nanoparticles were prepared by the sonochemical synthesis approach as reported in our previous report [15]. A schematic illustration of the preparation of MnFe_2_O_4_ spinel ferrite nanoparticles by the sonochemical synthesis approach is shown in Figure 1. Further, the synthesis condition for the preparation of these MnFe_2_O_4_ nanoparticles by the sonochemical method is tabulated in Table 1. For the preparation, manganese nitrate and iron nitrate was mixed with deionized water in a beaker. This solution was stirred on a magnetic stirrer for 5 min at room temperature. To this prepared mixed solution, sodium hydroxide aqueous solution was added and the whole mixed solution was placed under sonication (Ultrasonic homogenizer UZ SONOPULS HD 2070 (Berlin, Germany) (frequency: 20 kHz and power: 70 W)) for 20 min. The precipitate was collected and then washed with deionized water and ethanol and finally dried at 40 °C. Further, the increased particle size MnFe_2_O_4_ spinel ferrite nanoparticles were prepared for sonication time 40 min, 60 min and 80 min. The reaction temperature was 65 °C, 74 °C, 85 °C and 93 °C, after sonication time 20 min, 40 min, 60 min and 80 min, respectively. The synthesized MnFe_2_O_4_ nanoparticles were designated as MF20, MF40, MF60 and MF80 related to different sonication times 20 min, 40 min, 60 min and 80 min, respectively. Further, graphene oxide was prepared by the modified Hummer’s method [16]. Furthermore, graphene oxide (GO) was converted into reduced graphene oxide (RGO) by utilizing vitamin C as a reducing agent.

### 2.3. Preparation of Nanocomposites

**Figure 2:**
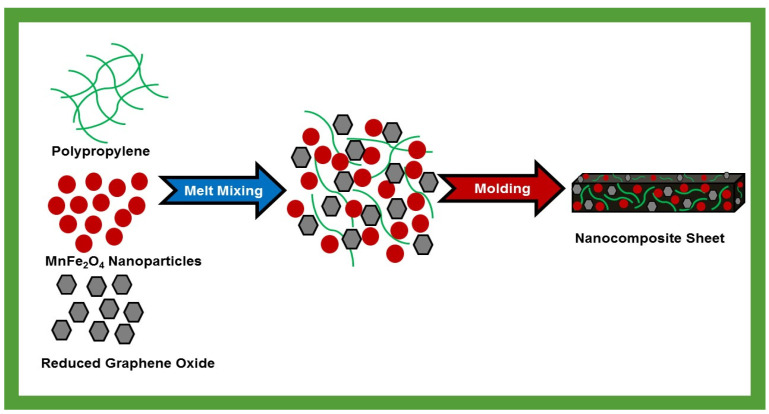
Schematic illustration of the preparation of polypropylene (PP) based nanocomposites embedded with MnFe_2_O_4_ spinel ferrite nanoparticles and reduced graphene oxide (RGO).

**Figure 3:**
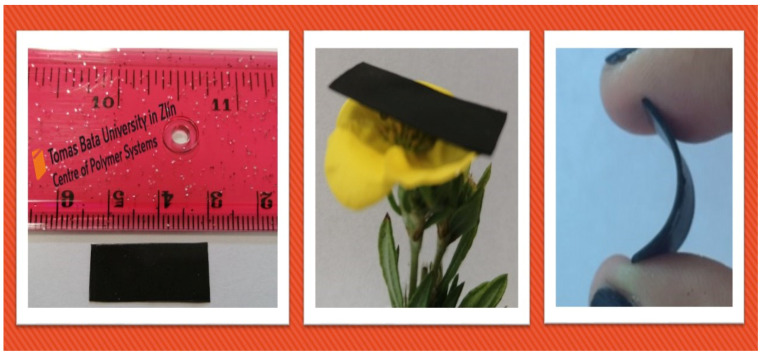
Digital photograph of PP nanocomposite embedded with MnFe_2_O_4_ spinel ferrite nanoparticles and reduced graphene oxide (RGO) as nanofillers.

A schematic illustration of the preparation of polypropylene (PP) based nanocomposites embedded with MnFe_2_O_4_ spinel ferrite nanoparticles and reduced graphene oxide (RGO) is shown in Figure 2. Nanocomposites of PP (50 wt %) with MnFe_2_O_4_ nanoparticles (40 wt %) and RGO (10 wt %) as nanofillers were developed by using the melt-mixing method. Four nanocomposite samples, namely (i) MF20-RGO-PP, (ii) MF40-RGO-PP, (iii) MF60-RGO-PP and (iv) MF80-RGO-PP were prepared. The rectangle-shaped sheet of a 22.86 × 10.16 × 0.5 mm^3^ dimension of prepared nanocomposites was developed by the hot-press approach. A representative digital photograph of PP nanocomposite embedded with MnFe_2_O_4_ spinel ferrite nanoparticles and reduced graphene oxide (RGO) as nanofillers is shown in Figure 3.

### 2.4. Characterization Techniques

The EMI shielding effectiveness of prepared nanocomposite (MF20-RGO-PP, MF40-RGO-PP, MF60-RGO-PP and MF80-RGO-PP) sheets of dimension 22.86 × 10.16 × 0.5 mm^3^ was studied with a vector network analyzer (Agilent N5230A) at 8.2–12.4 GHz (the so-called X-band) frequency range using a waveguide sample holder. X-ray powder diffraction (Rigaku Corporation, Tokyo, Japan) characterization tool was employed to analyze the crystal structure of nanocomposites. A field emission scanning electron microscope (FEI NanoSEM450) was employed to observe the morphology and presence of MnFe_2_O_4_ nanoparticles and reduced graphene oxide in the polypropylene matrix. Raman spectrometer (Thermo Fisher Scientific, Waltham, MA, USA) was used for Raman spectra of prepared RGO, PP, and its nanocomposites. A vibrating sample magnetometer (VSM 7407, Lake Shore) was employed to study magnetic hysteresis curves of prepared nanocomposite (MF20-RGO-PP, MF40-RGO-PP, MF60-RGO-PP and MF80-RGO-PP). The FTIR spectrometer (Nicolet 6700, Thermo Scientific) was utilized to achieve the FTIR spectra of prepared nanocomposites. Thermogravimetric analyses of prepared nanocomposites were performed on a Setaram LabSys Evo with TG/DSC sensor in an atmosphere of air (heating ramp 5 °C min^−1^, up to 1000 °C, and air flow 60 mL min^−1^). Mechanical properties of prepared polypropylene based nanocomposites were measured on a Testometric universal-testing machine of type M 350–5CT (Testometric Co. Ltd., Rochdale, UK).

## 3. Results

### 3.1. XRD Study

**Figure 4:**
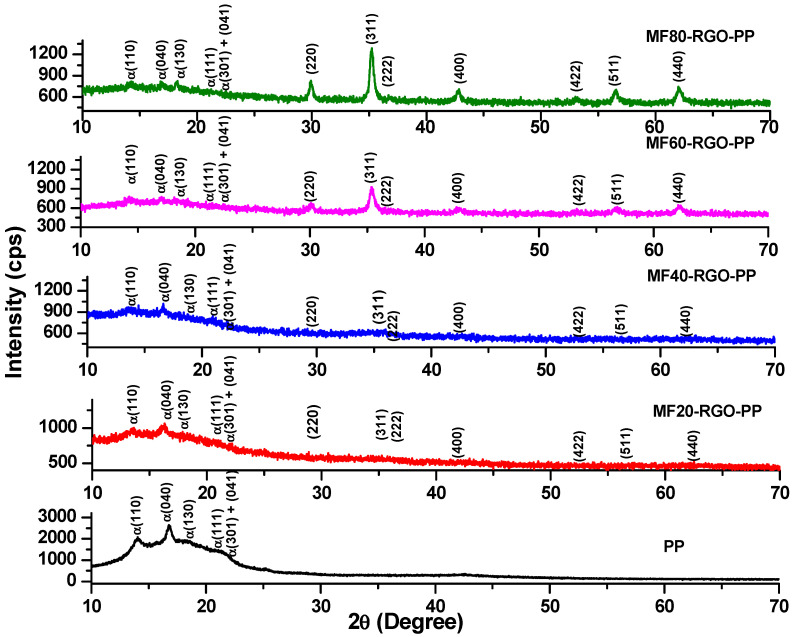
XRD pattern of polypropylene (PP) and prepared nanocomposites MF20-RGO-PP, MF40-RGO-PP, MF60-RGO-PP and MF80-RGO-PP.

XRD pattern of polypropylene (PP) and its prepared nanocomposites MF20-RGO-PP, MF40-RGO-PP, MF60-RGO-PP and MF80-RGO-PP is shown in Figure 4. The X-ray diffraction peaks indexed with (220), (311), (222), (400), (422), (511) and (440) confirm the presence of cubic spinel structure of MnFe_2_O_4_ nanoparticles in prepared nanocomposites [17]. It is noticeable in Figure 4 that the diffraction peak intensity of MnFe_2_O_4_ spinel ferrite nanoparticles was increased with the increase of sonication time, which signified an increase of crystallite size also [15]. The X-ray diffraction peaks at 14.2°, 16.8°, 18.2°, 21.1° and 21.9°, which is associated with (110), (040), (130), (111) and (131) + (041), respectively, crystal plane of the α-form of polypropylene [18]. Further, no diffraction peak associated with reduced graphene oxide was observed because of the low XRD intensity of RGO in prepared nanocomposites [19].

### 3.2. FE-SEM Study

**Figure 5:**
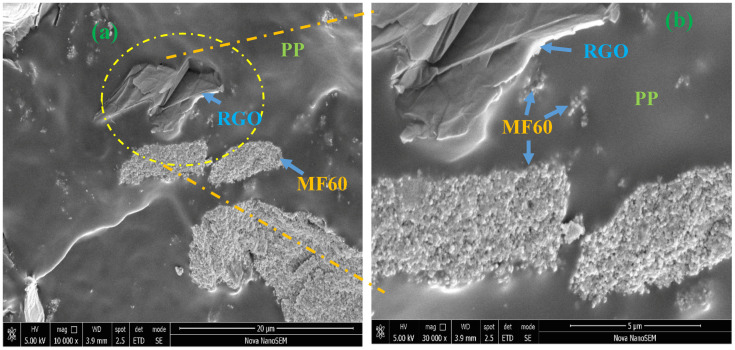

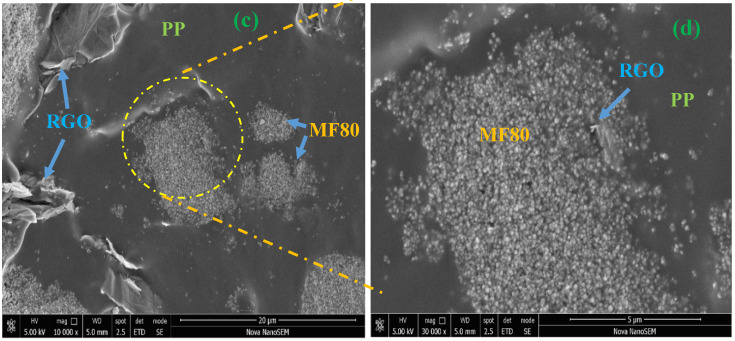
(**a**,**b**) FE-SEM image of cross-sections of the MF60-RGO-PP sample and (**c**,**d**) FE-SEM image of cross-sections of the MF80-RGO-PP sample.

**Figure 6:**
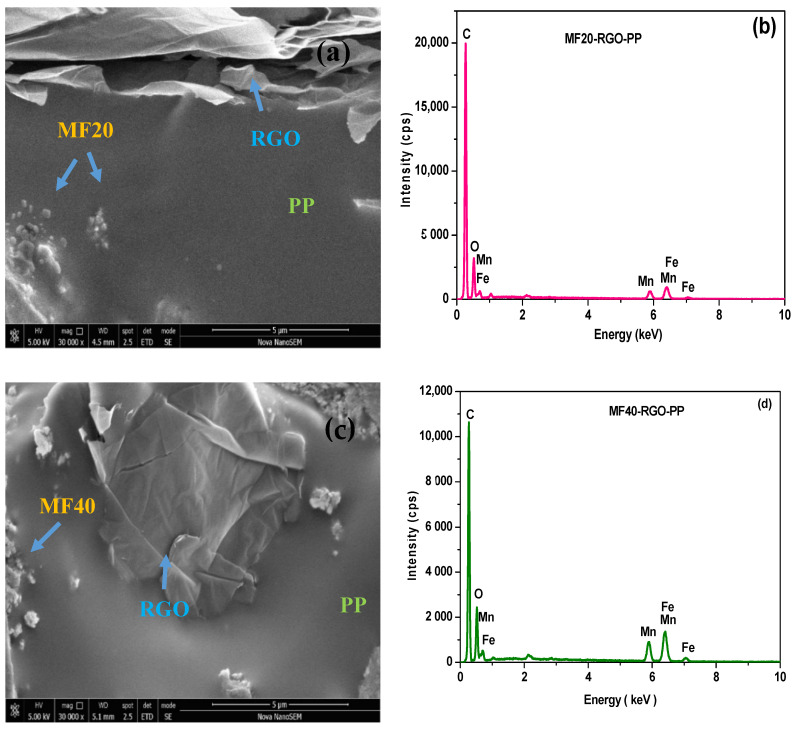
(**a**) FE-SEM image of cross-sections of MF20-RGO-PP, (**b**) EDX spectrum of MF20-RGO-PP, (**c**) FE-SEM image of cross-sections of MF40-RGO-PP and (**d**) EDX spectrum of MF40-RGO-PP.

Field emission scanning electron microscopy (FE-SEM) was utilized to investigate morphology of prepared nanocomposites. FE-SEM image of cross-sections of prepared MF60-RGO-PP and MF80-RGO-PP nanocomposites is shown in Figure 5. Images display the existence of MnFe_2_O_4_ spinel ferrite nanoparticles and reduced graphene oxide in the polypropylene matrix system. Further, FE-SEM image of prepared MF20-RGO-PP and MF40-RGO-PP nanocomposites is shown in Figure 6a,c, respectively. The presence of MnFe_2_O_4_ nanoparticles and reduced graphene oxide can be noticed in the polypropylene matrix. In addition, energy dispersive X-ray spectrum (EDX) of the MF20-RGO-PP (Figure 6b) and MF40-RGO-PP (Figure 6d) showed the existence of C, O, Mn and Fe.

### 3.3. Raman Spectroscopy

**Figure 7:**
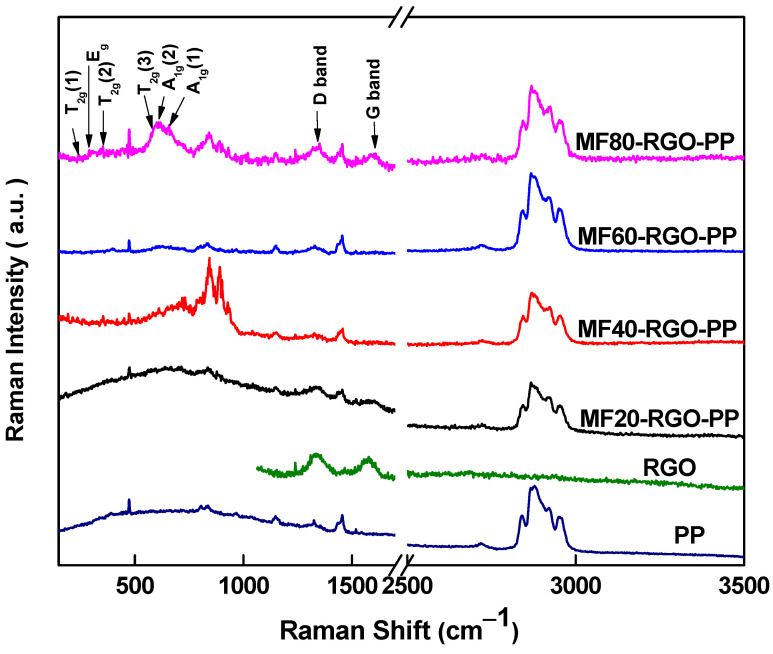
Raman spectrum of polypropylene (PP), reduced graphene oxide (RGO), MF20-RGO-PP, MF40-RGO-PP, MF60-RGO-PP and MF80-RGO-PP.

Figure 7 shows the Raman spectra of polypropylene (PP), reduced graphene oxide (RGO) and prepared nanocomposite MF20-RGO-PP, MF40-RGO-PP, MF60-RGO-PP and MF80-RGO-PP samples. The crystal structure and presence of MnFe_2_O_4_ in nanocomposites were confirmed through the measurement of A_1g_, E_g_ and T_2g_ peak positions in the Raman spectrum. In Figure 7, the existence of characteristics Raman bands, i.e., E_g_ mode (296 cm^−1^), T_2g_ mode (242 cm^−1^, 355 cm^−1^ and 580 cm^−1^) and A_1g_ mode (604 cm^−1^ and 657 cm^−1^) of spinel ferrite can be noticed [20]. The appearance of two characteristics peaks of RGO at 1338 cm^−1^ and 1594 cm^−1^ corresponds to the D-band and G-band of RGO, respectively [21]. Additionally, the other Raman peaks in the nanocomposites are associated with the chemical group of polypropylene [22].

### 3.4. FTIR Spectroscopy

**Figure 8:**
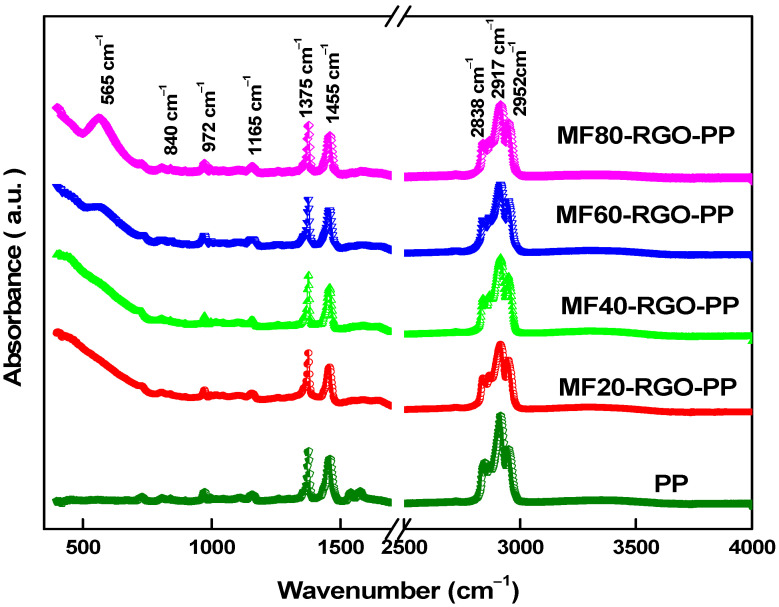
FTIR spectrum of polypropylene (PP), MF20-RGO-PP, MF40-RGO-PP, MF60-RGO-PP and MF80-RGO-PP.

Figure 8 displays the FTIR spectra of polypropylene (PP) and developed nanocomposite samples MF20-RGO-PP, MF40-RGO-PP, MF60-RGO-PP and MF80-RGO-PP. The presence of characteristic FTIR peaks of MnFe_2_O_4_ spinel ferrite nanoparticles and polypropylene can be noticed in the prepared nanocomposites, as shown in Figure 8. In spinel ferrite, the infrared bands noticed between 100 and 600 cm^−1^ indicate the formation of single phase spinel ferrite material. The absorption band at 565 cm^−1^ was associated with the intrinsic stretching vibration of metals at tetrahedral sites in MnFe_2_O_4_ nanoparticles [23]. The absorption peak at 840 cm^−1^ was associated with C–CH_3_ stretching vibration in PP. The peak 972 cm^−1^, and 1165 cm^−1^ were associated with –CH_3_ rocking vibration. The absorption peak at 1375 cm^−1^ and 2952 cm^−1^ were related to symmetric bending vibration of the –CH_3_ group and –CH_3_ asymmetric stretching vibration. The absorption peak at 1455 cm^−1^, 2838 cm^−1^ and 2917 cm^−1^ were related to –CH_2_-symmetric bending, –CH_2_-symmetric stretching, and –CH_2_-asymmetric stretching, respectively [24]. In amalgamation with Raman and FTIR spectroscopy results, the presence of MnFe_2_O_4_ spinel ferrite nanoparticles and reduced graphene oxide (RGO) in the polypropylene (PP) were confirmed.

### 3.5. Thermogravimetric Analysis (TGA)

**Figure 9:**
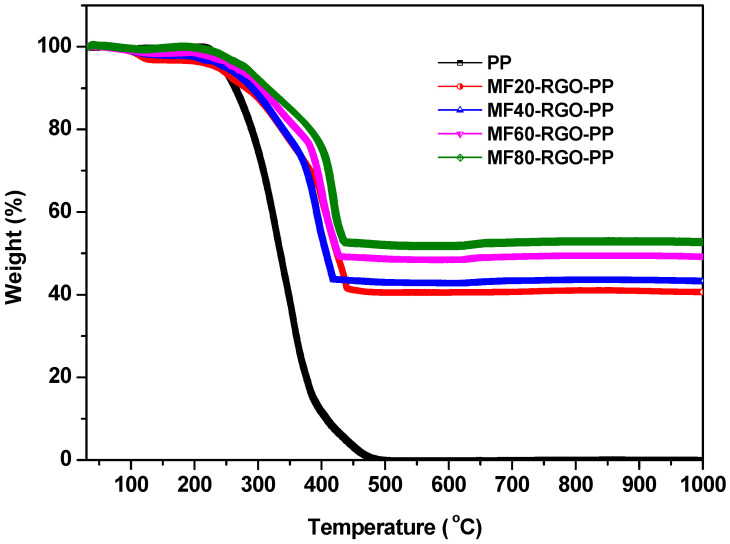
TGA curves of polypropylene (PP) and its prepared MF20-RGO-PP, MF40-RGO-PP, MF60-RGO-PP and MF80-RGO-PP nanocomposites under air atmosphere.

Figure 9 depicts the TGA curves of polypropylene (PP) and its prepared MF20-RGO-PP, MF40-RGO-PP, MF60-RGO-PP and MF80-RGO-PP nanocomposites under air atmosphere. It can be noticed that the PP had lower degradation temperature in comparison with its prepared nanocomposites. Further, nanocomposites exhibited higher thermal stability as compared to PP, which is associated with the result of an interaction between PP, MnFe_2_O_4_ nanoparticles and RGO [25]. Furthermore, the oxidative residues at 1000 °C are 37%, 39%, 46% and 49.2% for MF20-RGO-PP, MF40-RGO-PP, MF60-RGO-PP and MF80-RGO-PP, respectively, with 50% nanofillers loading [26]. The slightly lower residue values especially for MF20-RGO-PP and MF40-RGO-PP sample than the corresponding actual residues (i.e., loaded nano-fillers) were mainly due to the evaporation of surface impurities/chemical functional group attached on surface of small sized nanoparticles MF20 and MF40 [27].

### 3.6. Magnetic Property

**Figure 10:**
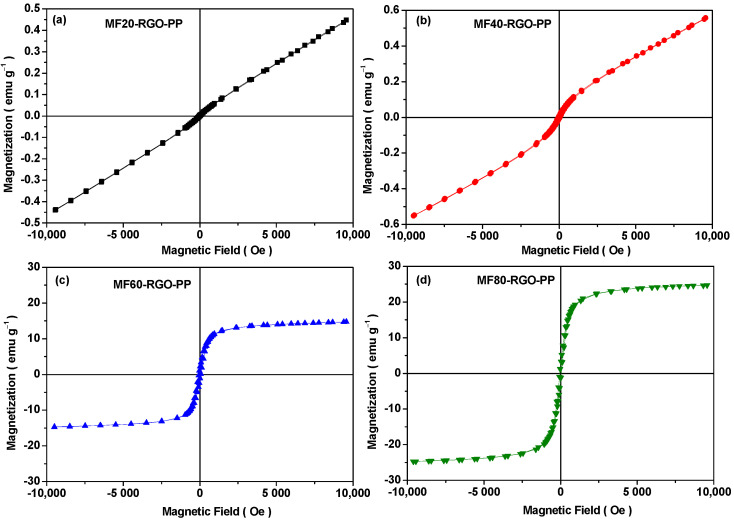
Magnetic hysteresis curves of prepared nanocomposites MF20-RGO-PP, MF40-RGO-PP, MF60-RGO-PP and MF80-RGO-PP samples.

Magnetic properties of prepared MF20-RGO-PP, MF40-RGO-PP, MF60-RGO-PP and MF80-RGO-PP nanocomposites were investigated by using a vibrating sample magnetometer. The magnetic hysteresis curves of MF20-RGO-PP, MF40-RGO-PP, MF60-RGO-PP and MF80-RGO-PP nanocomposites are shown in Figure 10. Ferromagnetic behavior can be noticed in magnetic hysteresis curves as depicted in Figure 10 for MF20-RGO-PP (*H_c_* = 33.2 Oe, *M_r_* = 0.003 emu/g, *M_s_* = 0.45 emu/g), MF40-RGO-PP (*H_c_* = 43.57 Oe, *M_r_* = 0.008 emu/g, *M_s_* = 0.55 emu/g), MF60-RGO-PP (*H_c_* = 61.0 Oe, *M_r_* = 1.57 emu/g, *M_s_* = 14.6 emu/g) and MF80-RGO-PP (*H_c_* = 45.9 Oe, *M_r_* = 2.03 emu/g, *M_s_* = 24.8 emu/g) nanocomposites. The ferromagnetic behavior of nanoparticles MF20 (*M_s_* = 1.9 emu/g, *H_c_* = 45.0 Oe, *M_r_* = 0.12 emu/g), MF40 (*M_s_* = 2.5 emu/g, *H_c_* = 42.0 Oe, *M_r_* = 0.13 emu/g), MF60 (*M_s_* = 30.2 emu/g, *H_c_* = 34.0 Oe, *M_r_* = 2.27 emu/g) and MF80 (*M_s_* = 52.5 emu/g, *H_c_* = 32.0, *M_r_* = 4.50 emu/g) was noticed, as mentioned in our previous report [15]. The high-frequency resonance in terms of anisotropy constant (*K*), anisotropy energy (*H_a_*) and resonance frequency (*f_r_*) has the following interrelationship with coercivity (*H_c_*) and saturation magnetization (*M_s_*) [28]:

(1) $$K=\frac{{µ}_{o}M_{s}H_{c}}{2}$$

(2) $$H_{a}=\frac{4\left|K\right|}{3{µ}_{o}M_{s}}$$

(3) $$2\pi f_{r}=rH_{a}$$

where *µ_o_* is the universal value of permeability in free space (4*π* × 10^−7^ H/m) and r is the gyromagnetic ratio. The correlation of the above equations signifies that the value of *H_c_* and *M_s_* can influence the magnitude of *K*, *H_a_* and *f_r_* and consequently electromagnetic properties of nanocomposites [29].

### 3.7. Electromagnetic Interference Shielding Effectiveness

The electromagnetic interference (EMI) shielding effectiveness (SE) is the degree of the material’s capability to block the electromagnetic waves. It is represented by the logarithm of the ratio of incident power (P_I_) to transmitted power (P_T_) in decibels

(4) $$SE_{T}\left(\mathrm{dB}\right)=10\log\left(\frac{P_{I}}{P_{T}}\right)$$

The attenuation of the electromagnetic waves involves generally three mechanisms: reflection (*SE_R_*), absorption (*SE_A_*) and multiple reflections (*SE_M_*). When the shielding effectiveness associated with absorption has a higher value than 10 dB, i.e., approximately all the rereflected waves will be absorbed within the material, the contribution associated with multiple reflections can be neglected [30]. Then, the total shielding effectiveness (*SE_T_*) can be expressed as

(5) $$SE_{T}=SE_{R}+SE_{A}$$

A two-port network analyzer can be utilized to measure the scattering parameters (*S*_11_, *S*_12_, *S*_21_ and *S*_22_), which correlates with reflection (*R*) and transmission coefficients (*T*) as [31]:

(6) $$T={\left|S_{12}\right|}^{2}={\left|S_{21}\right|}^{2}$$

(7) $$R={\left|S_{11}\right|}^{2}={\left|S_{22}\right|}^{2}$$

The shielding effectiveness due to absorption (*SE_A_*) and reflection (*SE_R_*) can be expressed in terms of the scattering parameters as

(8) $$SE_{R}=10\log\left(\frac{1}{1-R}\right)=10\log\left(\frac{1}{1-{\left|S_{11}\right|}^{2}}\right)$$

(9) $$SE_{A}=10\log\left(\frac{1-R}{T}\right)=10\log\left(\frac{1-{\left|S_{11}\right|}^{2}}{{\left|S_{21}\right|}^{2}}\right)$$

Therefore, total shielding effectiveness (*SE_T_*) can be obtained from the above relations as

(10) $$SE_{T}=20\log\left(S_{21}\right)$$

**Figure 11:**
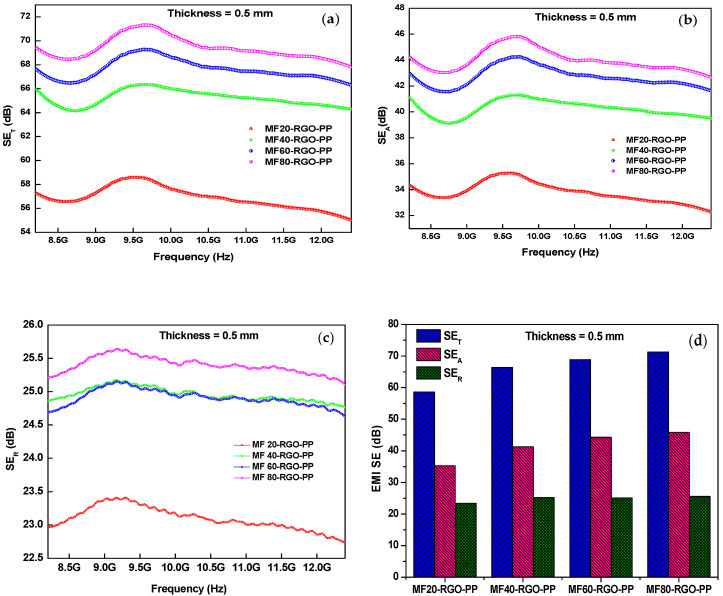
Electromagnetic interference (EMI) shielding effectiveness (**a**) *SE_T_*, (**b**) *SE_A_*, (**c**) *SE_R_* and (**d**) comparison plot of *SE_T_*, *SE_A_* and *SE_R_* for prepared nanocomposites MF20-RGO-PP, MF40-RGO-PP, MF60-RGO-PP and MF80-RGO-PP at a thickness of 0.5 mm.

Figure 11 depicts the EMI shielding effectiveness of prepared nanocomposites MF20-RGO-PP, MF40-RGO-PP, MF60-RGO-PP and MF80-RGO-PP at a thickness of 0.5 mm. The maximum value of total shielding effectiveness (*SE_T_*) was 58.6 dB, 66.4 dB, 69.4 dB and 71.3 dB for MF20-RGO-PP, MF40-RGO-PP, MF60-RGO-PP and MF80-RGO-PP samples, respectively, as shown in Figure 11a. Further, the maximum value of shielding effectiveness due to absorption (*SE_A_*) was 35.3 dB, 41.3 dB, 44.3 dB and 45.8 dB for MF20-RGO-PP, MF40-RGO-PP, MF60-RGO-PP and MF80-RGO-PP samples, respectively, as shown in Figure 11b. Additionally, the maximum value of shielding effectiveness due to reflection (*SE_R_*) was 23.4 dB, 25.2 dB, 25.1 dB and 25.6 dB for MF20-RGO-PP, MF40-RGO-PP, MF60-RGO-PP and MF80-RGO-PP samples, respectively, as shown in Figure 11c. The maximum value of total EMI SE (*SE_T_*), absorption (*SE_A_*) and reflection (*SE_R_*) of developed nanocomposites were plotted in Figure 11d. The results imply an absorption dominant shielding mechanism instead of reflection in the designed nanocomposites.

A research group, X.-J. Zhang et al. [32] noticed the minimum reflection loss -29.0 dB at 9.2 GHz for RGO/MnFe_2_O_4_/PVDF composites, which contained 5 wt % filler content with a thickness of 3.0 mm. P. Yin et al. [33] observed the optimal microwave absorbing intensity −48.92 dB at 0.78 GHz at a 2.5 mm thickness for the Apium-derived biochar loaded with MnFe_2_O_4_@C. Another researcher, R. V. Lakshmi et al. [34] observed a total shielding effectiveness value 44 dB in the X band frequency range for PMMA modified MnFe_2_O_4_-polyaniline nanocomposites. R. K. Srivastava et al. [35] noticed the total shielding effectiveness of −38 dB filler 5 wt % RGO-MnFe_2_O_4_ and 3 wt % of MWCNTs in polyvinylidene fluoride (PVDF) matrix. Another research group, Y. Wang et al. [36] observed the maximum reflection loss of −32.8 dB at 8.2 GHz with the thickness of 3.5 mm for MnFe_2_O_4_/RGO composite. Further, P. Yin et al. [37] noticed the maximum reflection loss of −14.87 dB at 2.25 GHz with the thickness of 4 mm. Furthermore, Y. Wang et al. [38] showed the maximum absorption of -38 dB at 6 GHz with the thickness of 3.5 mm for a ternary composite of Ag/MnFe_2_O_4_/reduced graphene oxide (RGO). Additionally, a comparison of electromagnetic wave absorption performance between MnFe_2_O_4_ spinel ferrite nanoparticles based developed composites reported in recent years are tabulated in Table 2.

**Figure 12:**
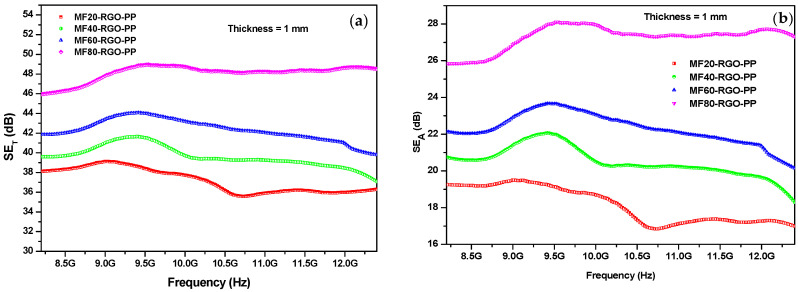

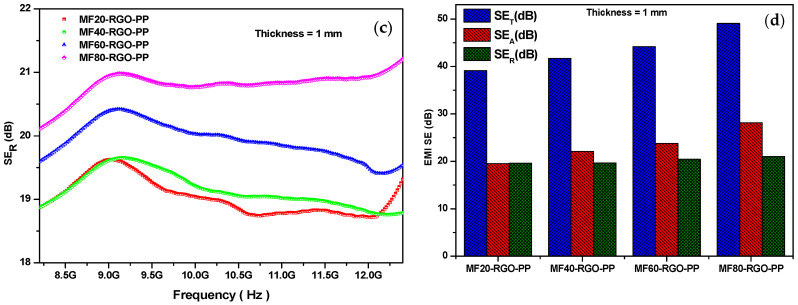
EMI shielding effectiveness (**a**) *SE_T_*, (**b**) *SE_A_*, (**c**) *SE_R_* and (**d**) comparison plot of *SE_T_*, *SE_A_* and *SE_R_* for MF20-RGO-PP, MF40-RGO-PP, MF60-RGO-PP and MF80-RGO-PP nanocomposite sheet at thickness of 1 mm.

The influence of thickness on EMI shielding effectiveness of prepared nanocomposites was also investigated. Figure 12 depicts EMI shielding effectiveness of prepared nanocomposites MF20-RGO-PP, MF40-RGO-PP, MF60-RGO-PP and MF80-RGO-PP nanocomposite sheet at a thickness of 1 mm. The maximum value of total shielding effectiveness (*SE_T_*) was 39.15 dB, 41.72 dB, 44.21 dB and 49.11 dB for MF20-RGO-PP, MF40-RGO-PP, MF60-RGO-PP and MF80-RGO-PP nanocomposite sheet, respectively, at a thickness of 1 mm, as depicted in Figure 12a. Further, the maximum value of shielding effectiveness due to absorption (*SE_A_*) was 19.52 dB, 22.07 dB, 23.78 dB and 28.12 dB for MF20-RGO-PP, MF40-RGO-PP, MF60-RGO-PP and MF80-RGO-PP nanocomposite, respectively, at a thickness of 1 mm, as shown in Figure 12b. Furthermore, the maximum value of shielding effectiveness due to reflection (*SE_R_*) was 19.64 dB, 19.67 dB, 20.44 dB and 21.00 dB for MF20-RGO-PP, MF40-RGO-PP, MF60-RGO-PP and MF80-RGO-PP nanocomposite, respectively, at a thickness of 1 mm, as represented in Figure 12c. A comparative value of *SE_T_*, *SE_A_* and *SE_R_* for prepared nanocomposite sheet at a thickness of 1 mm is represented in Figure 12d. A slight decrease in shielding effectiveness was noticed with an increase in thickness from 0.5 to 1 mm [39,40], however, the observed value of EMI shielding effectiveness was higher enough than the limit (20 dB) needed for techno-commercial applications [41].

### 3.8. Electromagnetic Properties and Parameters

**Figure 13:**
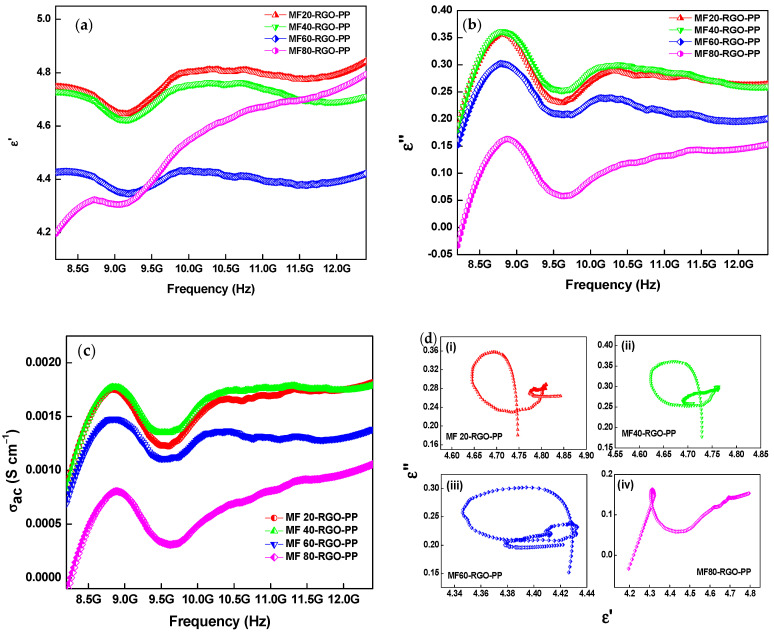
Frequency-dependent (**a**) real permittivity (*ε*’), (**b**) imaginary permittivity (*ε*″), (**c**) ac conductivity (*σ*_ac_) and (**d**) Cole–Cole semicircles (*ε*′ vs. *ε*′′) of prepared MF20-RGO-PP, MF40-RGO-PP, MF60-RGO-PP and MF80-RGO-PP composite samples.

It is well-known that the electromagnetic interference shielding performances of the nanocomposites are highly associated with complex permittivity ($\varepsilon_{r}=\varepsilon^{\prime }+j\varepsilon^{\prime \prime }$) and complex permeability ($\mu_{r}=\mu^{\prime }+j\mu^{\prime \prime }$). Figure 13a shows the real part of the complex permittivity (*ε*’) of prepared nanocomposites. The real part of the complex permittivity (*ε*’) corresponds to the storage of the electrical energy and can be controlled by polarization in the material. The existence of RGO and MnFe_2_O_4_ nanoparticles in the PP matrix created a heterogeneous medium that acted as interface accumulation in the developed nanocomposites. The value of the real part of the complex permittivity (*ε*’) was in the range of 4.64–4.84, 4.62–4.76, 4.34–4.42 and 4.20–4.79 for MF20-RGO-PP, MF40-RGO-PP, MF60-RGO-PP and MF80-RGO-PP sample, respectively. The real part of the permittivity (*ε*’) was associated with the polarization in the material, which consisted of dipolar polarization, interfacial/surface polarization, orientational polarization, ionic or electronic polarization. The higher value of the real part of permittivity (*ε*’) of MF20-RGO-PP and MF40-RGO-PP was due to presence of a higher number of surface impurity bonds/residual bonds and cluster defects in MF20 and MF40 nanoparticles via a chemical synthesis route, the electrons were not evenly distributed, which led to orientation polarization and thereby further enhancement in the real part of permittivity [42]. The ionic or electronic polarization plays a dominant role in enhancing the real part of permittivity at a high frequency. The existence of this polarization enhanced slowly with increase of frequency. Therefore, the increase of real part of permittivity (*ε*’) at higher frequency in MF80-RGO-PP sample could be associated with dominant role of electronic polarization [43]. The real part of the complex permittivity (*ε*’) had no direct relation with the total shielding effectiveness (*SE_T_*). The imaginary part of the permittivity (*ε*″) corresponded to the dielectric loss in the materials. Figure 13b depicts the imaginary part of the permittivity (*ε*″) of the developed nanocomposites. It can be observed that the value of the imaginary part of the complex permittivity (*ε*″) was in the range of 0.18–0.35, 0.17–0.36, 0.15–0.30 and −0.03–0.15 for MF20-RGO-PP, MF40-RGO-PP, MF60-RGO-PP and MF80-RGO-PP nanocomposite, respectively.

According to free-electron theory, the electrical conductivity (*σ*_ac_) can be evaluated by the following relation [44]:

(11) $$\sigma_{ac}=\varepsilon_{o}\varepsilon^{\prime \prime }\omega =\varepsilon_{o}\varepsilon^{\prime \prime }2\pi f$$

where *σ*_ac_, *ε*_o_, ω and f are the electrical conductivity, the dielectric constant of the free space, the angular frequency and frequency of the electromagnetic waves, respectively. Figure 13c depicts the frequency dependence variation of the electrical conductivity (*σ*_ac_) of the developed nanocomposites. The value of electrical conductivity was 9.04 × 10^−4^ to 1.84 × 10^−3^ S/cm, 8.41 × 10^−4^ to 1.80 × 10^−3^ S/cm, 7.25 × 10^−4^ to 1.47 × 10^−3^ S/cm and −8.55 × 10^−5^ to 1.05 × 10^−3^ S/cm for prepared MF20-RGO-PP, MF40-RGO-PP, MF60-RGO-PP and MF80-RGO-PP composite samples, respectively. The MF20 and MF40 nanoparticles-based nanocomposites had similar electrical conductivity and also higher than the other two nanoparticles MF60 and MF80 based nanocomposites. The enhanced electrical conductivity is associated with an increased induced microcurrent network and hopping phenomenon in prepared nanocomposites [45,46].

The relative complex permittivity can be expressed by the following relation [47]:

(12) $$\varepsilon_{r}=\varepsilon_{\infty }+\frac{\varepsilon_{s}-\varepsilon_{\infty }}{1+j2\pi f\tau }\;=\varepsilon^{\prime }-j\varepsilon^{\prime \prime }$$

where *f*, *ε*_∞_, *ε*_s_ and *τ* corresponds to frequency, optical and stationary dielectric constant and polarization relaxation time, respectively. The dielectric parameters (*ε*’, *ε*″) can be evaluated by the following relation [48,49]:

(13) $$\varepsilon^{\prime }=\varepsilon_{\infty }+\frac{\varepsilon_{s}-\varepsilon_{\infty }}{1+\omega^{2}\tau^{2}}\;$$

(14) $$\varepsilon^{\prime \prime }=\varepsilon_{r}^{\prime \prime }+\varepsilon_{c}^{\prime \prime }=\frac{\varepsilon_{s}-\varepsilon_{\infty }}{1+\omega^{2}\tau^{2}}+\frac{\sigma }{\omega \varepsilon_{o}}$$

where *σ*, $\varepsilon_{r}^{\prime \prime }$ and $\varepsilon_{c}^{\prime \prime }$ corresponds to electrical conductivity, polarization loss and conductive loss, respectively.

The dielectric loss is generally associated with Debye polarization relaxation, which includes ionic polarization, electron polarization, dipole polarization and interfacial polarization [50]. The interfacial polarization originated because of the heterogeneous interfaces between MnFe_2_O_4_ nanoparticles and reduced graphene oxide in the polypropylene matrix. Based on Debye theory, the polarization characteristics can be confirmed by Cole–Cole semicircles, which is resulting from the following relation [51]:

(15) $${\left(\varepsilon^{\prime }-\frac{\varepsilon_{s}-\varepsilon_{\infty }}{2}\right)}^{2}+{\left(\varepsilon^{\prime \prime }\right)}^{2}={\left(\frac{\varepsilon_{s}-\varepsilon_{\infty }}{2}\right)}^{2}$$

Figure 13d depicts the Cole–Cole semicircles (*ε*′ vs. *ε*″) of prepared MF20-RGO-PP, MF40-RGO-PP, MF60-RGO-PP and MF80-RGO-PP composite samples. Several semicircles can be noticed for prepared nanocomposites, which indicate the coexistence of multiple polarization process. Further, since the prepared MnFe_2_O_4_-RGO-PP composites can simply form conductive networks and therefore conduction loss cannot be ignored also.

**Figure 14:**
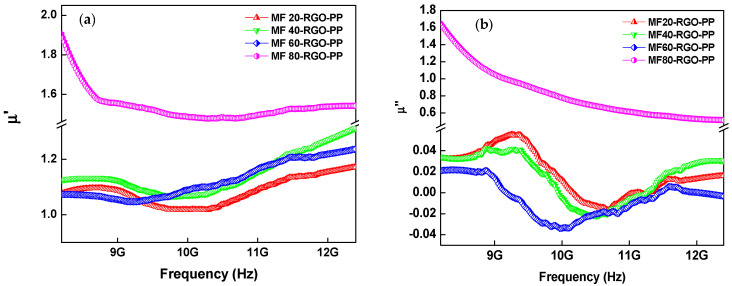

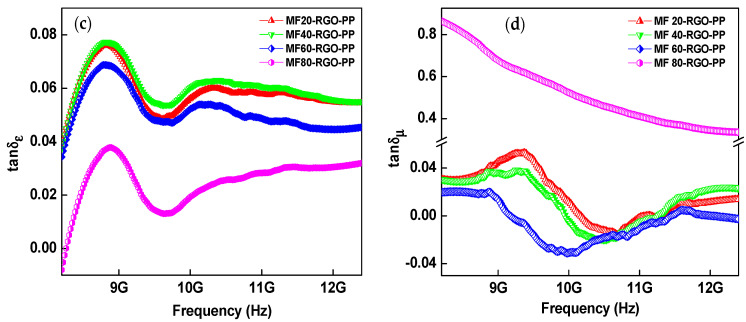
Frequency-dependent (**a**) real part of permeability (*µ*’), (**b**) imaginary part of permeability (*µ*″), (**c**) dielectric loss (tan*δ_ε_*) and (**d**) magnetic loss (tan*δ_µ_*) of prepared MF20-RGO-PP, MF40-RGO-PP, MF60-RGO-PP and MF80-RGO-PP composite samples.

In general, the real permeability (*μ*’) signifies the energy storage capacity of magnetic energy and the imaginary permeability (*μ*″) refers to the energy dissipation capacity of magnetic energy [52]. Figure 14a depicts the frequency dependence variation of the real part of permeability (*µ*’) of designed nanocomposites. The value of the real part of permeability (*µ*’) was 1.01–1.17, 1.06–1.31, 1.04–1.23 and 1.48–1.89 for the prepared MF20-RGO-PP, MF40-RGO-PP, MF60-RGO-PP and MF80-RGO-PP, respectively. Noticeably, the higher value of the real part of permeability (*µ*’) of the MF80-RGO-PP sample suggesting the increased storage capacity of magnetic energy in comparison to other prepared nanocomposites. Figure 14b shows the frequency dependence variation of the imaginary part of permeability (*µ*″) of developed nanocomposite samples. The value of the imaginary part of permeability (*µ*″) was −0.015–0.057, −0.024–0.042, −0.033–0.023 and 0.52–1.62 for the prepared MF20-RGO-PP, MF40-RGO-PP, MF60-RGO-PP and MF80-RGO-PP, respectively. The negative value of imaginary permeability in the frequency range 9–11.4 GHz for MF60-RGO-PP, 10–11.2 GHz for MF40-RGO-PP and 10.2–10.7 GHz for MF20-RGO-PP can be associated with the eddy current caused by an extra magnetic field, which nullifies the inherent magnetic field [53]. Based on the Maxwell equations, the negative values of the imaginary part of permeability signify that the magnetic energy is radiated out and converted into electric energy [54].

Additionally, the dielectric loss tangent (tan*δ_ε_* = *ε*″/*ε*’) was utilized to calculate the loss capability against the stored capacity for electric energy. Figure 14c depicts the frequency dependence variation of dielectric loss (tan*δ_ε_*) of developed nanocomposites. Noteworthy, the trend of value of dielectric loss (tan*δ_ε_*) of developed nanocomposites was similar to the trend of the imaginary part of permittivity (*ε*″). The value of dielectric loss (tan*δ_ε_*) was 0.042–0.075, 0.037–0.077, 0.035–0.068 and −0.008–0.038 for prepared MF20-RGO-PP, MF40-RGO-PP, MF60-RGO-PP and MF80-RGO-PP composite samples, respectively.

Moreover, the magnetic loss ability of the prepared nanocomposites can be evaluated by the magnetic tangent loss (tan*δ_μ_* = *μ*″/*μ*’). Figure 14d depicts the frequency dependence changes in magnetic loss (tan*δ_μ_*) of prepared nanocomposites. The value of magnetic loss (tan*δ_μ_*) was −0.014–0.053, −0.021–0.037, −0.031–0.021 and 0.331–0.853 for prepared MF20-RGO-PP, MF40-RGO-PP, MF60-RGO-PP and MF80-RGO-PP, respectively.

Generally, the magnetic loss is attributed to the magnetic resonance (natural resonance and exchange resonance), eddy current loss, magnetic hysteresis loss and domain wall resonance [55]. The magnetic hysteresis loss had no appearance in the weak electromagnetic field, whereas the domain wall resonance had occurrence only at 1–100 MHz. The magnetic resonance and the eddy current effect induced the magnetic loss in the range of GHz frequency. When the magnetic loss was associated with the eddy current loss, the value of *μ*″(*μ*’)^−2^*f*^−1^ should be constant with the variation of the frequency [56].

**Figure 15:**
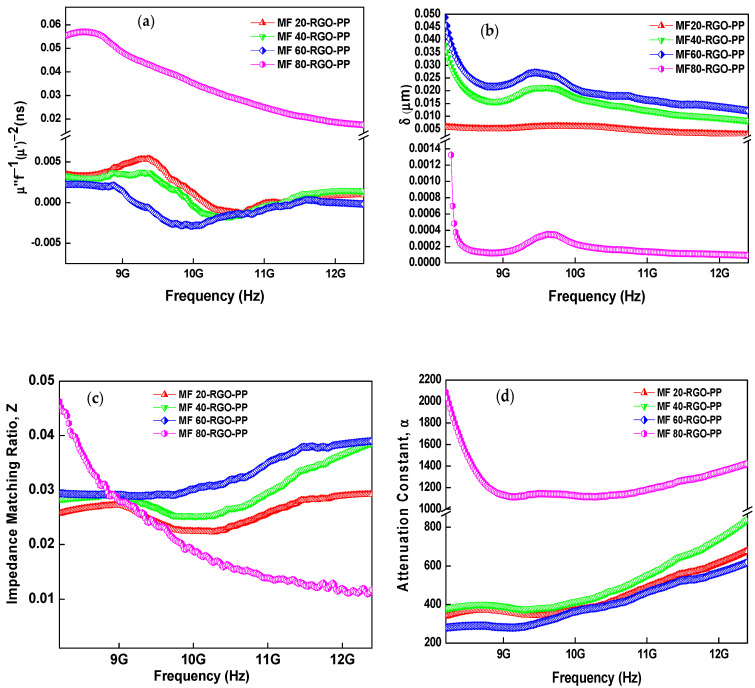
Frequency-dependent (**a**) eddy current loss, (**b**) skin depth, (**c**) impedance matching ratio and (**d**) attenuation constant of prepared MF20-RGO-PP, MF40-RGO-PP, MF60-RGO-PP and MF80-RGO-PP composite samples.

The eddy current can be calculated by using the following relation [57]:

(16) $$C_{o}=\;\mu^{’’}{\left(\mu ’\right)}^{-2}f^{-1}=2\pi \sigma \mu_{o}d^{2}/3$$

where *μ_o_* is the permeability of the vacuum, *σ* is the electric conductivity and d is the thickness of the material. As shown in Figure 15a, the value of C_o_ was constant at a lower frequency range from 8.2 to 8.8 GHz for MF20-RGO-PP, MF40-RGO-PP and MF60-RGO-PP nanocomposites, which implies that the magnetic loss in this frequency range was eddy current loss. Further, for these nanocomposites, the value of *μ*″(*μ*’)^−2^*f*^−1^ varied at a higher frequency from 8.8 to 12.4 GHz, which suggests that the magnetic loss was not only induced by eddy current effect but also natural ferromagnetic resonance. Furthermore, for MF80-RGO-PP composite sample, the value of *μ*″(*μ*’)^−2^*f*^−1^ was not constant throughout the whole frequency range.

The superior value of EMISE was associated with the low skin depth of the prepared nanocomposites. The skin depth (*δ*) is the depth where the incident power of the EM waves fell to 1/e of its value at the surface. It can be given by the following relation [58]:

(17) $$\delta ={\left(\pi f\mu \sigma \right)}^{-1/2}$$

where *f* is the frequency, *μ* is the permeability of the material and *σ* is the electrical conductivity. Figure 15b depicts the variation of skin depth (*δ*) of prepared MF20-RGO-PP, MF40-RGO-PP, MF60-RGO-PP and MF80-RGO-PP composite samples. The skin depth varied from 0.003 to 0.007 μm, 0.008 to 0.036 μm, 0.012 to 0.048 μm and 0.0012 to 0.0013 μm for MF20-RGO-PP, MF40-RGO-PP, MF60-RGO-PP and MF80-RGO-PP nanocomposites, respectively. It was noticed that the value of the skin depth of nanocomposites was much lower than their thickness, which leads to a high EMI SE [59]. Further, in general, the material with the shallowest skin depth exhibits high absorption loss [60].

In general, to achieve a large role of electromagnetic absorption, the shielding material should exhibit a large impedance matching ratio (Z) to free space [61]. The impedance matching ratio (Z) can be evaluated from the following relation [62]:

(18) $$Z=Z_{1}/Z_{o}={\left(\mu_{r}/\varepsilon_{r}\right)}^{1/2}$$

where, *Z*_1_ is the impedance matching of the electromagnetic wave absorber material, and *Z_o_* is the impedance in free space. As shown in Figure 15c, the impedance matching ratio was increased with the increase of nanoparticle size of MnFe_2_O_4_ spinel ferrite in developed MF20-RGO-PP, MF40-RGO-PP and MF60-RGO-PP nanocomposites, whereas it was increased more at a lower frequency and decreased more at a higher frequency in case of the MF80-RGO-PP nanocomposite sample.

The other important electromagnetic parameter for electromagnetic interference shielding nanocomposites is the electromagnetic wave attenuation, and the attenuation constant (*α*) can be evaluated by the following relation [63]:

(19) $$\alpha =\frac{\sqrt{2}\pi f}{c}{\left[\left({µ}^{\prime \prime }\varepsilon^{\prime \prime }-{µ}^{\prime }\varepsilon^{\prime }\;\right)+\;\sqrt{{\left({µ}^{\prime \prime }\varepsilon^{\prime \prime }-{µ}^{\prime }\varepsilon^{\prime }\right)}^{2}+{\left({µ}^{\prime }\varepsilon^{\prime \prime }+\;{µ}^{\prime \prime }\varepsilon^{\prime }\right)}^{2}}\right]}^{1/2}$$

**Figure 16:**
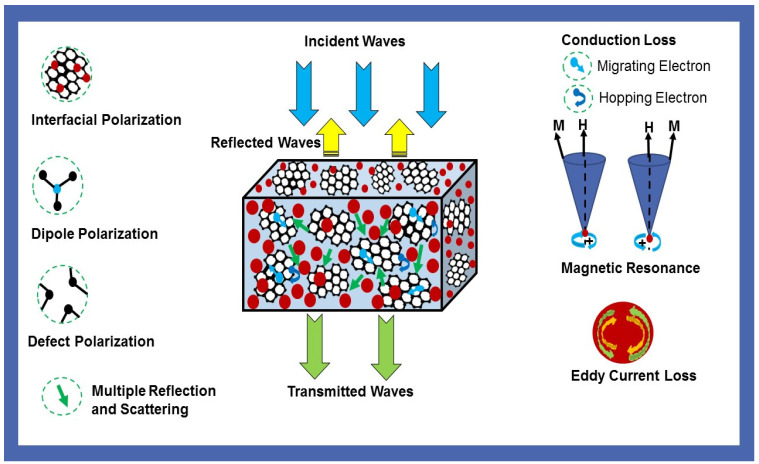
Schematic illustration of the electromagnetic interference shielding mechanism in prepared nanocomposites.

Figure 15d depicts the attenuation constant of prepared MF20-RGO-PP, MF40-RGO-PP, MF60-RGO-PP and MF80-RGO-PP composite samples. In general, a large value of attenuation constant (*α*) indicates a good attenuation ability, which reveals the great dissipation characteristics of materials [64]. It can be observed that the nanocomposites MF20-RGO-PP, MF40-RGO-PP and MF60-RGO-PP had a very similar attenuation constant (*α*) value in the frequency range 8.2–12.4 GHz, whereas there was a noticeable gap in the attenuation constant (*α*) value of MF80-RGO-PP composite samples, especially in the low-frequency range. These results indicate that the total loss ability of MF80 spinel ferrite nanoparticles based nanocomposites displayed high magnetic loss in comparison with other samples. Moreover, a clear design of the electromagnetic wave shielding mechanism as reflected above is illustrated in Figure 16.

### 3.9. Mechanical Properties

**Figure 17:**
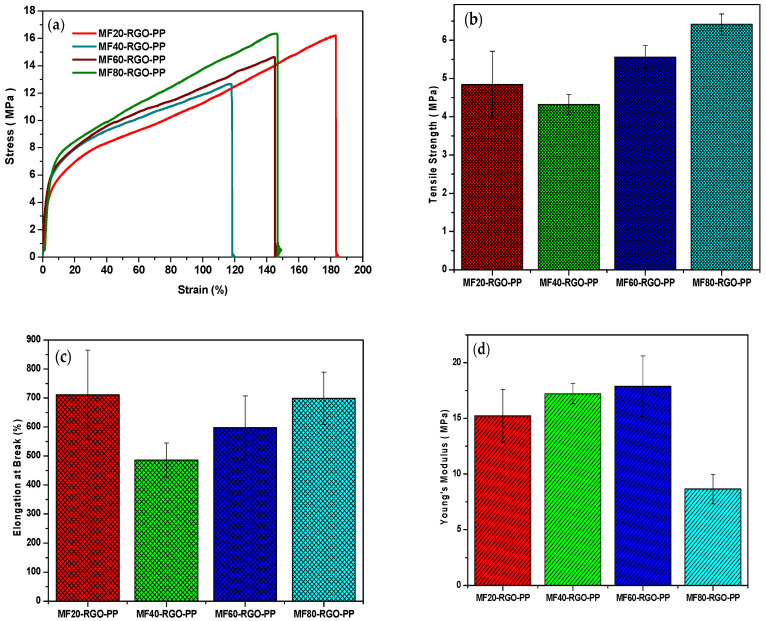
Mechanical behavior of prepared MF20-RGO-PP, MF40-RGO-PP, MF60-RGO-PP and MF80-RGO-PP nanocomposites: (**a**) representative strain–stress curves, (**b**) the tensile strength, (**c**) elongation at break and (**d**) Young’s modulus.

In general, the variation in mechanical properties is associated with particle size, morphology and loading amount of fillers in polymer matrix [65,66]. Figure 17a depicts representative strain-stress curves of prepared MF20-RGO-PP, MF40-RGO-PP, MF60-RGO-PP and MF80-RGO-PP nanocomposites. The extracted mechanical parameter tensile strength of prepared nanocomposites is depicted in Figure 17b. The value of tensile strength was 4.84 MPa, 4.32 MPa, 5.56 MPa and 6.42 MPa for MF20-RGO-PP, MF40-RGO-PP, MF60-RGO-PP and MF80-RGO-PP, respectively. Further, Figure 17c depicts the extracted mechanical parameter elongation at break for prepared MF20-RGO-PP, MF40-RGO-PP, MF60-RGO-PP and MF80-RGO-PP nanocomposites. The value of elongation at break was 711%, 486%, 598% and 699% for MF20-RGO-PP, MF40-RGO-PP, MF60-RGO-PP and MF80-RGO-PP, respectively. Furthermore, the extracted mechanical parameters Young’s modulus is shown in Figure 17d. The value of Young’s modulus was 15.2 MPa, 17.2 MPa, 17.8 MPa and 8.7 MPa for MF20-RGO-PP, MF40-RGO-PP, MF60-RGO-PP and MF80-RGO-PP, respectively.

## 4. Conclusions

We developed electromagnetic interference shielding nanocomposites based on polypropylene (PP) matrix with reduced graphene oxide (RGO) and MnFe_2_O_4_ spinel ferrite nanoparticles as nanofillers. Different sized magnetic filler MnFe_2_O_4_ (namely MF20, MF40, MF60 and MF80 samples) nanoparticles were prepared by the sonochemical approach at sonication synthesis time 20, 40, 60 and 80 min. It was noticed that the electromagnetic interference shielding performances of designed nanocomposites MF20-RGO-PP, MF40-RGO-PP, MF60-RGO-PP and MF80-RGO-PP were also controlled with the tuning of dielectric/magnetic loss. The maximum value of total shielding effectiveness (SE_T_) was 71.3 dB for MF80-RGO-PP nanocomposite with a thickness of 0.5 mm in the frequency range (8.2–12.4 GHz). The excellent electromagnetic interference shielding properties with a lightweight, flexible and thinness sheet of developed nanocomposites was realized.

## Figures and Tables

**Table 1:** Synthesis condition for preparation of MnFe_2_O_4_ nanoparticles by the sonochemical method.

Sample	Concentration of Mn (NO_3_)_2_ 4H_2_O	Concentration of Fe (NO_3_)_2_ 9H_2_O	Concentration of NaOH	Sonication Time	Reaction Temperature
**MF20**	0.17 M	0.36 M	1.66 M	20 min	65 °C
**MF40**	0.17 M	0.36 M	1.66 M	40 min	74 °C
**MF60**	0.17 M	0.36 M	1.66 M	60 min	85 °C
**MF80**	0.17 M	0.36 M	1.66 M	80 min	93 °C

**Table 2:** Comparison of electromagnetic wave absorption performance between MnFe_2_O_4_ spinel ferrite nanoparticles based developed composites reported in recent years.

No.	Shielding Material	Frequency (GHz)	Specimens Thickness (mm)	Effect of Shielding	Ref.
1.	RGO/MnFe_2_O_4_/PVDF	2–18 GHz	3.0 mm	−29.0 dB	[32]
2.	Biochar/MnFe_2_O_4_@C	0.2–3 GHz	2.5 mm	−48.92 dB	[33]
3.	PMMA modified MnFe_2_O_4_-PANI	8–12 GHz		~44 dB	[34]
4.	PVDF/RGO-MnFe_2_O_4_/MWCNTs	8–18 GHz		−38dB	[35]
5.	MnFe_2_O_4_/RGO	2–18 GHz	3.5 mm	−32.8 dB	[36]
6.	SiO_2_-MnFe_2_O_4_	0.2–3 GHz	4 mm	−14.87 dB	[37]
7.	Ag/MnFe_2_O_4_/RGO	2–18 GHz	3.5 mm	−38 dB	[38]
8.	MnFe_2_O_4_-RGO-PP	8.2–12.4 GHz	0.5 mm	~71.3 dB	This work
